# Cholelithiasis increased prostate cancer risk: evidence from a case–control study and a meta-analysis

**DOI:** 10.1186/s12894-022-01110-8

**Published:** 2022-10-03

**Authors:** Ya-Dong Li, Zheng-Ju Ren, Liang Gao, Jun-Hao Ma, Yuan-Qing Gou, Wei Tan, Chuan Liu

**Affiliations:** Department of Urology, The Second Affiliated Hospital, Chongqing Medical University, Chongqing, China

**Keywords:** Prostate cancer (PCa), Cholelithiasis, Meta-analysis, Cholecystectomy

## Abstract

**Introduction:**

Cholelithiasis represents a known risk factor for digestive system neoplasm. Few studies reported the association between cholelithiasis and the risk of prostate cancer (PCa), and the results were controversial.

**Methods:**

We reviewed the medical records of the Second Affiliated Hospital of Chongqing Medical University Hospital to perform a retrospective matched case–control study, which included newly diagnosed 221 PCa patients and 219 matched controls. Logistic regression was applied to compare cholelithiasis exposure and adjusted for confounding factors. Additionally, we conducted a meta-analysis pooling this and published studies further to evaluate the association between cholelithiasis and PCa risk. Related ratio (RR) and 95% confidence interval (95%CI) were used to assess the strength of associations.

**Results:**

Our case–control study showed that cholelithiasis was associated with a higher incidence of PCa (OR = 1.87, 95% CI: 1.06–3.31) after multivariable adjustment for covariates. The incidence of PCa was increased in patients with gallstones but not cholecystectomy. 7 studies involving 80,403 individuals were included in the meta-analysis. Similarly, the results demonstrated that cholelithiasis was associated with an increased risk of PCa (RR = 1.35, 95%CI: 1.17–1.56) with moderate-quality evidence. Cholelithiasis patients with low BMI increased the PCa incidence. Moreover, Subgroup analysis based on region showed that cholelithiasis was associated with PCa in Europe (RR = 1.24, 95%CI 1.03–1.51) and Asia (RR = 1.32, 95%CI 1.24–1.41).

**Conclusions:**

The results suggested an association between cholelithiasis and the risk of PCa. There was no significant relationship between cholecystectomy therapy and PCa risk. Further cohort studies should be conducted to demonstrate the results better.

## Introduction

Worldwide more than 1,275,000 men are diagnosed annually with PCa. PCa is the second most common malignancy and the leading cause of cancer-associated mortality in men [1]. PCa could be seen in America and Europe, but little is known about its etiology [2, 3]. Established risk factors for PCa include age, ethnicity, germline mutation (BRCA5.35%, ATM1.6%, and CHEK1.9%), and dietary factors [4–7]. Due to the heterogeneity and the multiple-factor of PCa, it is crucial to identify more risk factors.

With the prevalence of about 5–15% of the western population, cholelithiasis was an important public health problem in Europe and America [8]. Cholelithiasis has many risk factors, including genetic, environmental risk factors, diabetes, and metabolic syndrome, although hypersecretion of cholesterol plays a vital role in promoting the formation of gallstones [9]. The mechanism suggested that high cholesterol or metabolic syndrome may have tumorigenesis on the digestive system neoplasm and PCa. High cholesterol is associated with the development of PCa and can play an essential role in tumorigenesis by accumulating in cancer tissues [10, 11]. Consistent with these findings, men who take statins after prostatectomy would have less possibility of becoming aggressive PCa [12, 13]. The association between metabolic syndrome and the risk of developing PCa is emerging [8, 14, 15]. In addition, men with gallstones have disorders of gut microbiota, which may lead to prostate carcinogenesis [16, 17]. Recently, emerging epidemiological evidence suggested that previous cholelithiasis was correlated with the development and procession of PCa [18–20].

The relationship between the two diseases is still controversial. Given the potential risk factor of cholelithiasis in PCa, we assessed the relationship between PCa and cholelithiasis and conducted a hospital-based case–control study in the Second Affiliated Hospital of Chongqing Medical University Hospital, China. Furthermore, we performed a systematic meta-analysis of published studies and our case–control study to evaluate the association between cholelithiasis and the risk of PCa.

## Methods

### Case–control study

This retrospective case–control was performed in the second affiliated hospital of Chongqing medical university study to investigate the association between cholelithiasis and PCa risk. Based on the confirmed pathological diagnosis record after performing a diagnostic biopsy or operation, 221 patients with newly diagnosed PCa were included in this study between 2018 and 2020. The patients with a history of malignancies or a family history of cancer were excluded. The 219 matched controls were randomly included from the patients admitted to the same hospitals. These controls were non-malignant neoplastic conditions unrelated to known risk factors for PCa. All the subjects were Chinese.

According to subjects’ electronic medical records, we collected relative information, including a history of cholelithiasis, lifestyle habits (including tobacco smoking and alcohol consumption), and comorbidity. The patients with a history of cholelithiasis were proven to provide the following information through subjects’ electronic medical records: abdominal ultrasound/ computed tomography, the presence of a relevant scar or having a clear medical record. If subjects with cholelithiasis were diagnosed more than 12 months before PCa diagnosis for cases or controls, we would consider them to have pre-existing cholelithiasis. Naturally, they would be excluded if they provided a date of the previous cholelithiasis no more than 12 months or lacked medical records. The ethics committee approved this study of the Second Affiliated Hospital of Chongqing Medical University following the ethical standards of the institutional and/or national research committee and with the 1964 Helsinki declaration. We did not require informed consent from the patients because we collected data by reviewing medical records.

## Meta-analysis

### Literature search and study inclusion criteria

Following the PRISMA guidelines, the literature search of Medline, EMBASE, and Web of Science was conducted up to 1 November 2021. The following keywords or Medical Subject Heading (MeSH) terms related to PCa and cholelithiasis were used, including “Prostate Neoplasms, Prostate Neoplasm, Prostatic Neoplasm, Prostate cancer, Prostatic Cancer” and Risk factors and “Cholelithiasis, Gallstones or cholecystectomy.” There were no limitations on the language of studies to be included. The reference lists of the retrieved studies were manually searched for additional studies. Studies would be included if they met the following inclusion criteria: (a) these studies should be the associations of cholelithiasis with PCa risk; (b) these studies must be observational; (c) these studies provide risk estimates with 95% corresponding confidence intervals (CIs) were available. Reviews, case reports, and studies with overlapping or unavailable data were all excluded. All disagreements were resolved by discussion.

### Data extraction

Two authors (YDL and ZJR) independently extracted the following data from the included studies: author, publication date, country or region, study design, follow-up time, sample size, the number of PCa cases, risk estimates with corresponding 95% Cis, and adjusted factors. These data were extracted using a predefined data extraction sheet.

### Quality assessment

Two independent authors used the Newcastle–Ottawa scale (NOS) to perform quality assessment of included studies. Each study had three aspects: selection of participants, study comparability, and outcome evaluation [21]. The NOS scores with9, 7–8 and ≤ 6 were separately considered high, intermediate, or low in each study.

### Grading the quality of evidence

The levels of evidence for outcomes were performed based on the GRADEpro approach (https://gradepro.org/), which assessed the aspects of risk of bias, inconsistency, indirectness, imprecision of the results, and publication bias. The evidence levels included very low, low, moderate, or high.

### Statistical analysis

In the case–control study, we analyzed the category variables using the chi-squared test and the continuous variables using an independent sample t test, respectively. Then, we assessed the association between cholelithiasis and PCa risk using the odds ratio (OR) and its corresponding 95% confidence interval (CI) by performing unconditional logistic regression models with or without adjusting for age and lifestyle habits, and comorbidity. We further explored the association between cholecystectomy or gallstones and PCa risk. All data were analyzed using SPSS 17.0, and the *P* value of < 0.05 with two-tailed tests indicated significance.

For the meta-analysis, the association of cholelithiasis with PCa risk was measured by pooling the risk estimate. According to the study design, region, cholecystectomy, or gallstones, subgroup analyses of the primary outcomes were performed. The summary of effects for the outcomes was calculated as risk ratio (RR) and 95% confidence intervals (CI) using a random effect model. Both χ2-based Q test and I2 test were performed to estimate the between-study heterogeneity. *P* < 0.05 and I2 > 50% were regarded to be statistically significant for the between-study heterogeneity. Based on the between-study heterogeneity, we use a fixed or random effect model. Publication bias was evaluated by using funnel plots and Begg’s and Egger’s tests. All statistical analyses were performed using Stata statistical software (ver.12.0, Stata, College Station, TX, USA). *P* value < 0.05 was considered to indicate statistical significance.

## Results

### Case–control study

221 cases were newly diagnosed PCa, and 219 controls were recruited. Table 1 shows the detailed characteristics of the participants. The average age of cases was 72.29 ± 8.02 years, and of controls were 71.14 ± 7.68 years with no statistical significance. Cases and controls had a consistent difference in smoking, drinking, and having a history of hypertension, stroke, COPD, and coronary artery disease. Additionally, compared to controls, cases were more likely to have a history of diabetes and cholelithiasis (*P* < 0.05).

**Table 1:** Characteristics of PCa patients and controls (China, 2018–2020)

Variable	Case(221)	Control(219)	*P* value
Mean (SD) Age at diagnosis	72.29 (8.02)	71.14 (7.68)	0.268
Smoking (%)			0.840
Yes	112 (50%)	113 (51%)	
No	109 (50%)	106 (49%)	
Alcohol drinking (%)			0.474
Yes	78 (36%)	86 (39%)	
No	141 (64%)	135 (61%)	
Diabetes (%)	39 (18%)	24 (11%)	0.045
Hypertension (%)	89 (40%)	79 (36%)	0.365
Coronary artery disease (%)	26 (12%)	18 (8%)	0.215
Stroke (%)	16 (7%)	13 (6%)	0.582
COPD (%)	12 (5%)	18 (8%)	0.246
Cholelithiasis (%)	40 (18%)	22 (10%)	0.015

Table 2 summarizes the association between cholelithiasis and PCa risk. The results revealed that cholelithiasis was significantly associated with a higher risk of PCa (OR = 1.98, 95%CI: 1.13–3.46) based on the crude OR estimated by the univariate analysis. After multivariable adjustment for confounding factors, the adjusted OR for increased risk of PCa patients with cholelithiasis was 1.87 (95%CI: 1.06, 3,31). Moreover, we explored the association between cholecystectomy or gallstones and PCa risk as a subgroup analysis. There was a significant association of gallstones with PCa, with a multivariate-adjusted OR of 2.53 (95% CI: 1.08–5.96). However, no association was observed between the history of cholecystectomy and PCa risk.

**Table 2:** Analysis of Odds ratios (OR) and 95% confidence intervals (CI) of prostate cancer for cholelithiasis

Prostate cancer	case	control	Crude OR(95%CI)	Adjusted OR (95%CI)^a^
No cholelithiasis	181	197	1 (ref)	1 (ref)
Cholelithiasis	40	22	1.98 (1.13–3.36)	1.87 (1.06–3,31)
Gallstones	21	7	2.77 (1.20–6.40)	2.53 (1.08–5.96)
Cholecystectomy	23	17	1.38(0.72–2.66)	1.25 (0.64–2.45)

^a^The OR and 95% CI has been adjusted for age, smoking alcohol drinking, hypertension, diabetes, stroke, COPD, coronary artery disease

## Meta-analysis

**Fig. 1:**
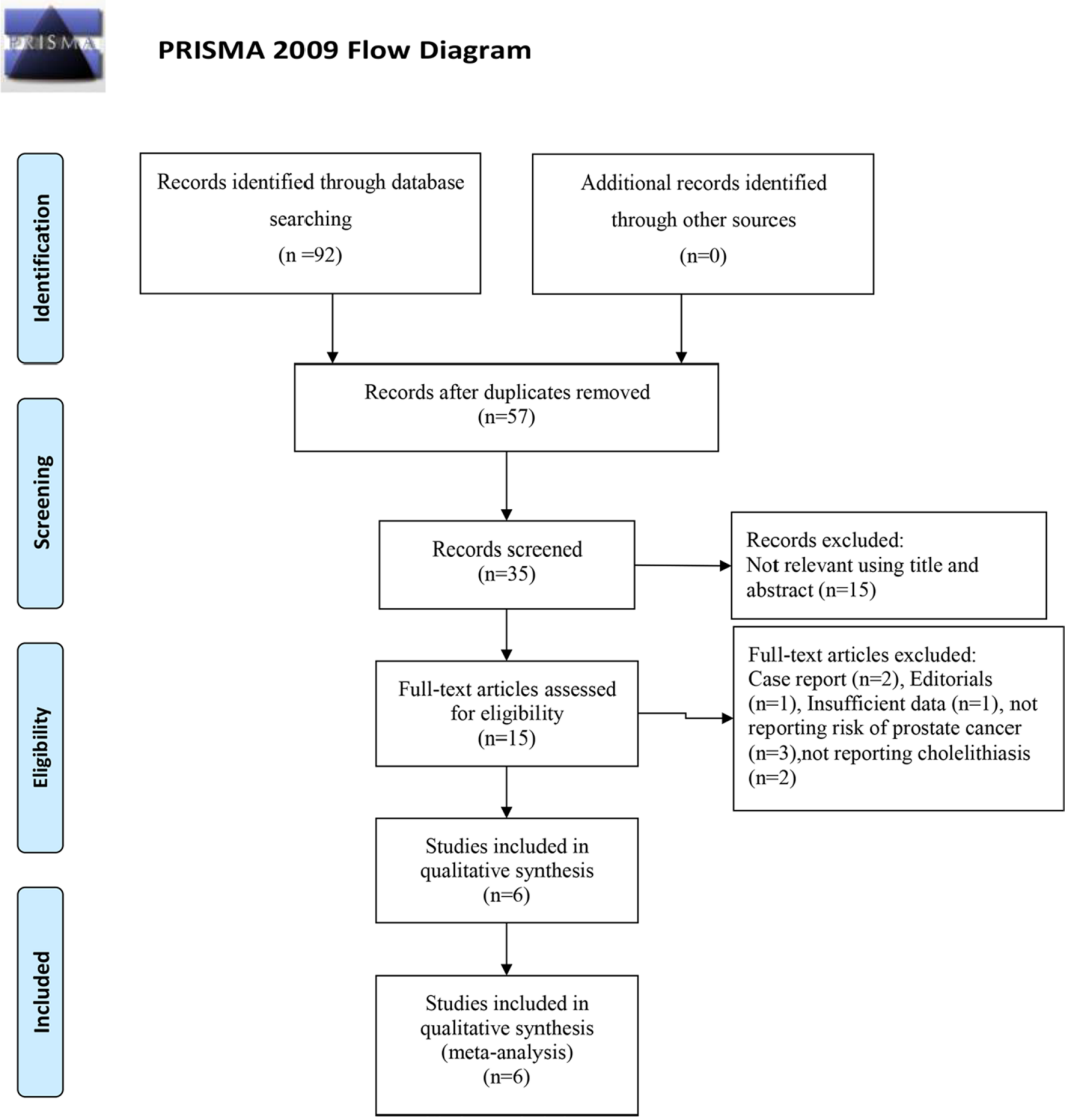
Flow chart showing this study selection

The detailed process of the literature search was showed in Fig. 1. Finally, 6 published studies were included in our analysis by evaluating full-text review (Fig. 1) [19, 20, 22–25]. Table 3 summarized the characteristics of included studies. Among those studies, there were 3 cohort studies and 3 case–control studies. 3 came from Europe, and 3 came from the Asia region. The publication date of these studies was from 2004 to 2017. 6 studies involving 3560 cases among 79,963 individuals investigated the associations between cholelithiasis and PCa risk. The quality of studies was summarized in Table 4. All eligible studies were defined as high quality (NOS ≥ 6) (Table 4).

**Table 3:** Characteristics of studies included in the meta-analysis

Author	Year	Country	Study disgn	Follow up duration	Sample size	Exposure	Measure of effect	RR(cholelithiasis risk) (95% CI)	Adjustment factors
A.Tavani	2011	Italy, Switzer-land	Case–control	–	Case: 1582 Control: 2231	History of cholelithiasis	OR	1.36(1.04,1.78)	Age, study center, year of interview, study period, education, alcohol drinking, tobacco smoking, and BMI
F.Bravi	2005	Italy	Case–control	–	Case: 1294 Control: 1451	History of gallstones	OR	1.26(0.93,1.70)	Age, center, education, BMI, physical activity, tobacco smoking, alcohol consumption and family history of prostate cancer
Qiang Li	2010	Japan	Cohort	1995–2003	22,458	History of cholelithiasis	HR	1.72(1.12–2.66)	Age, hypertension, family history of cancer education level, marital status, BMI, time, spent, walking, smoking status, alcohol drinking, total energy intake per day, consumption of green tea, daily consumption of calcium and daily consumption of fish and dairy products
Shabanzadeh	2017	Denmark	Cohort	1892–2014	3017	History of gallstone disease	HR	0.67(0.35,1.30)	Age, sex, cohort number, BMI, non-high density lipoprotein cholesterol, high density lipoprotein cholesterol, smoking alcohol consumption, diet, physical activity level, social group
Chien-Hua Chen	2016	Taiwan	Cohort	1998–2011	47,479	Gallbladder stone disease	HR	1.30(1.22,1.39)	Age, occupation, urbanization level, comorbidity of hyperlipidemia, diabetes, hypertension, BPH, urinary stones, urinary tract infection, obesity, asthma, CAD,COPD, stroke, and antihypertensive medications
Kim	2004	Koreans	Case–control	–	Case:184 Control: 267	History of cholelithiasis	OR	2.40(1.02,5.68)	–

**Table 4:** Quality assessment of included studies

References		Selection	Comparability	Exposure	Total
A.Tavani	2011	***	**	***	8
F.Bravi	2005	**	**	**	6
Qiang Li	2010	***	**	***	8
Shabanzadeh	2017	***	**	***	8
Chien-Hua Chen	2016	**	*	***	7
Kim	2004	**	**	**	6

**Fig. 2:**
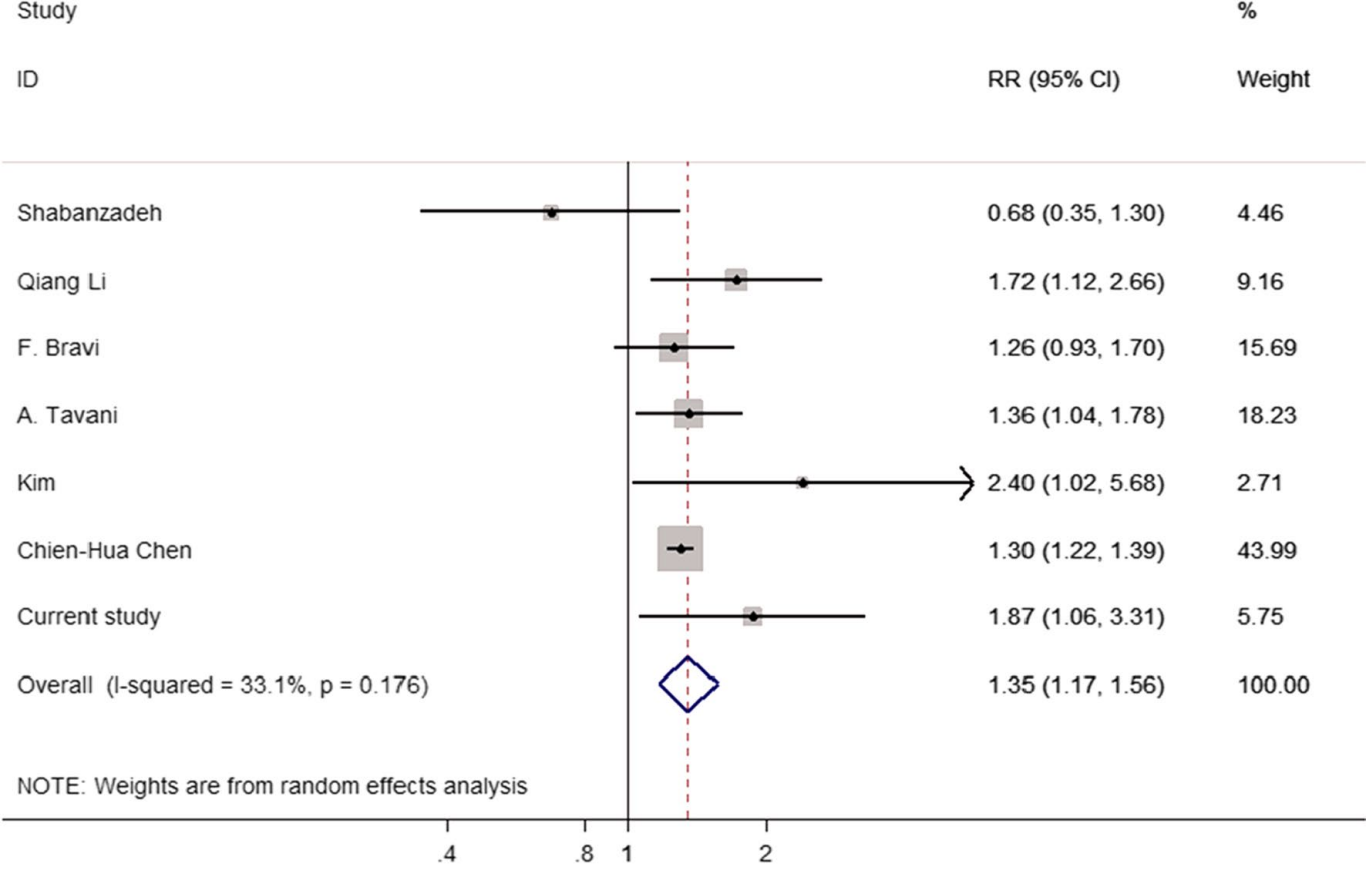
Forest plot including studies depicting pooling relative risk for developing prostate cancer

A total of 7 studies, including our studies and 6 published studies, were eligible in this meta-analysis. As shown in Fig. 2, the pooled RR of PCa for men with a history of cholelithiasis was 1.35(95%CI: 1.17–1.56), with moderate-quality evidence (Table 5). There was no obvious heterogeneity (I^2^ = 32.4%, *P* = 0.181) using a random effects model for assessment.

**Table 5:** GRADE assessment of quality of the body of evidence, and summary of findings

Association studied	No. of studies	Design	Risk of bias	Inconsistency	Indirectness	Imprecision	Factors that can increase quality of evidence	Pooled effect estimate	Quality
Cholelithiasia and risk of PCa	7	Observational study	Not serious	Not serious	Not serious	Not serious	All plausible confounding would reduce a demonstrated effect	1.35(1.17, 1.56)	⨁⨁⨁◯ MODERATE
Gallstones and risk of PCa	3	Observational study	Not serious	Not serious	Not serious	Not serious	None	1.16(0.55, 2.46)	⨁⨁◯◯ LOW
Cholecystectomy and risk of PCa	2	Observational study	Not serious	Not serious	Not serious	Not serious	None	1.27(0.74, 2.19)	⨁⨁◯◯ LOW

PCa prostate cancer

To better evaluate the association between cholelithiasis and PCa risk. We conducted a subgroup analysis based on the study regions, BMI level, gallstones and cholecystectomy. The results of the subgroup analysis were shown in Table 6. Stratified analysis among European and Asian showed an increased risk of PCa for men with a history of cholelithiasis (RR = 1.24, 95%CI: 1.03–1.51), (RR = 1.32, 95%CI: 1.24–1.41), comparing to men without cholelithiasis. Besides, cholelithiasis increased PCa risk, as suggested by the pooled RR of case–control studies, but not cohort studies. Men with cholelithiasis with lower BMI had a higher risk of PCa (RR = 1.54, 95%CI: 1.16–2.06), as suggested by stratified analyses by BMI level. Moreover, we observed that cholecystectomy or gallstones was not related to the risk of PCa.

**Table 6:** Subgroup analysis for studies included in the analysis

Variable	No. of studies	Pooled RR (95% CI)	I^2^ statistics (%)	*P* value for the heterogeneity Q test
Region				
Europe	3	1.24(1.03, 1.51)	45.70%	0.158
Asia	4	1.32(1.24, 1.41)	39.40%	0.176
Study design				
Case–control	4	1.40(1.17, 1.69)	0.70%	0.389
Cohort	3	1.25(0.89, 1.77)	0.00%	0.618
BMI		2.58(1.21, 5.54)	0.00%	0.472
low	3	1.54(1.16, 2.06)	0.00%	0.958
high	2	1.30(1.02, 1.66)	66.60%	0.052
Gallstones	3	1.18(0.55, 2.48)	73.10%	0.024
Cholecystectomy	2	1.27(0.74, 2.19)	0.00%	0.936

*BMI* Body mass index

**Fig. 3:**
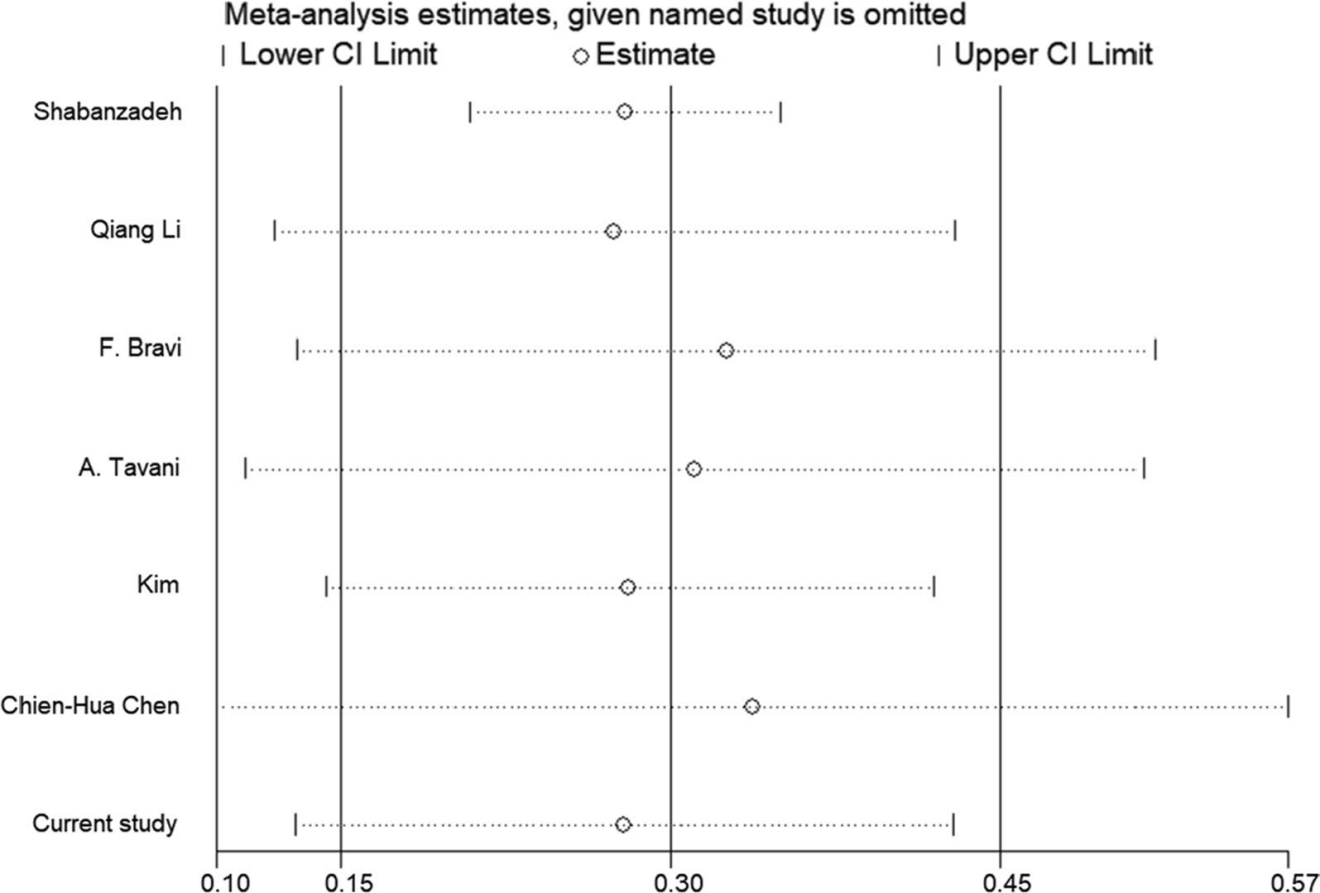
Sensitivity analysis investigates each study's influence on the overall risk of prostate cancer

**Fig. 4:**
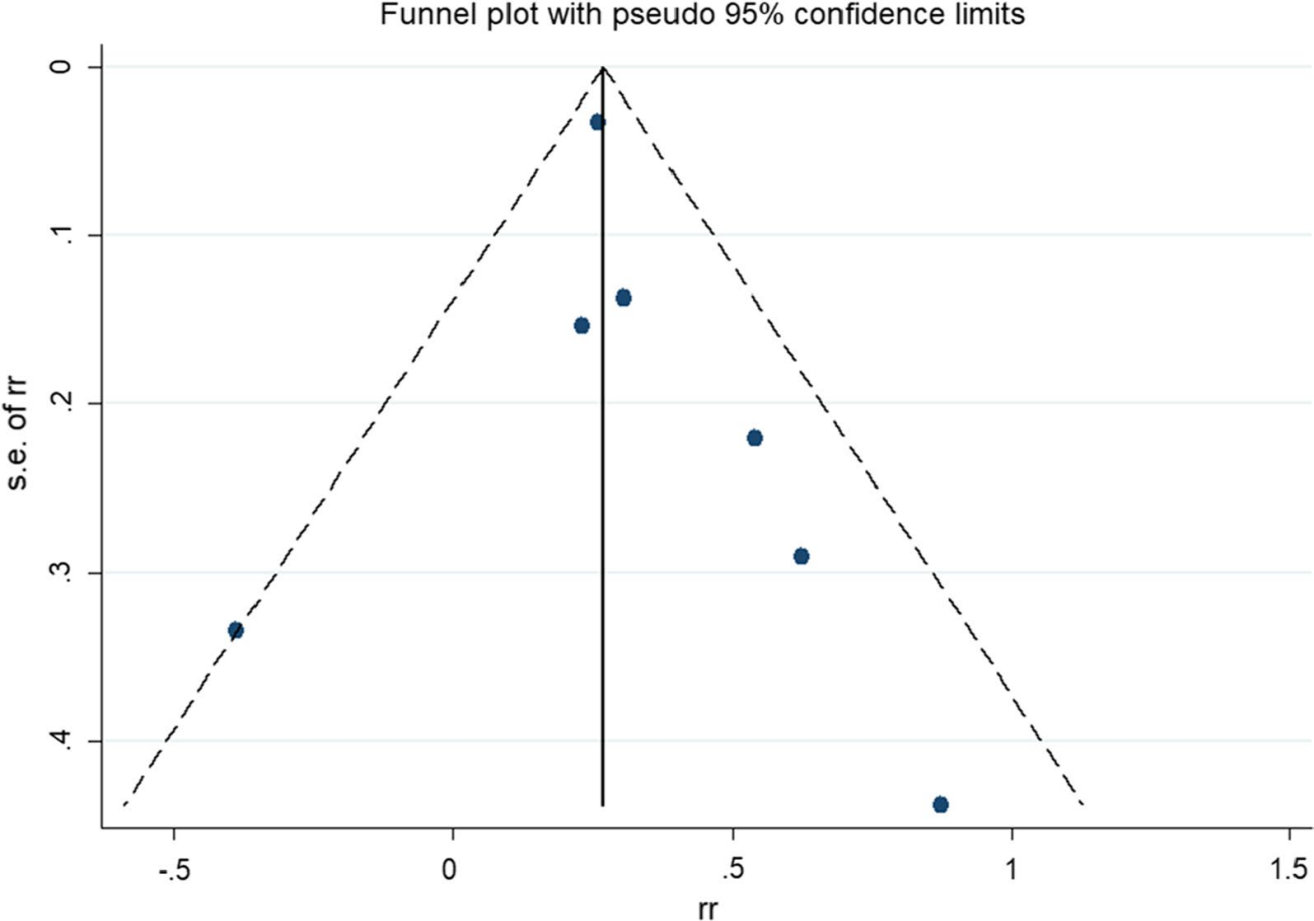
Funnel plot assessing publication bias about the association between cholelithiasis and the risk of prostate cancer

We performed a sensitivity analysis to evaluate the risk of cholelithiasis on the overall estimate by removing individual studies, and we observed the stability of the results of this meta-analysis (Fig. 3). Visual inspection of funnel plots showed no evident asymmetry (Fig. 4). Publication bias was not observed based on both Begg’s (*P* = 0.545) and Egger’s test (*P* = 0.368) for PCa risk.

## Discussion

This study aimed to evaluate the impact of cholelithiasis on the subsequent PCa risk. In our case–control study, our findings from 221 cases and 219 controls provided evidence that men with pre-existing cholelithiasis had an increased risk of PCa, whereas we did not observe a significant association between cholecystectomy and PCa risk. Combined with the results of our meta-analysis, the history of cholelithiasis was significantly associated with PCa risk. However, as for cholecystectomy or gallstones, no significant risk difference was observed.

Some previous studies have reported an association between a history of cholelithiasis and cardiovascular, cholangiocarcinoma and pancreatic cancer risk [18, 26, 27]. In older men, there was a higher risk of cholelithiasis. Whether cholelithiasis modified the risk of PCa is worth investigating by considerable studies. Bravi et al. [25] reported that finding from 1294 cases and 1451 controls demonstrated that men with gallstones did not seem to be associated PCa risk in a case–control study. Recently, Qiang Li et al. [20] found that men with cholelithiasis had a higher incidence of PCa compared without cholelithiasis, especially for advanced PCa in the Ohsaki cohort followed from 1995 to 2003. Due to these inconsistent findings, our study provided evidence from a case–control combined with a meta-analysis to estimate the underlying association between cholelithiasis and PCa susceptibility. The results supported that cholelithiasis is a risk factor for the development of PCa.

A total of 7 studies were included in this meta-analysis. And we found that men with a history of cholelithiasis were associated with PCa risk. The increased PCa risk was also seen for both European and Asian population, as suggested by subgroup analysis according to a different region. In addition, the increased risk of PCa was observed for cholelithiasis patients with high and low BMI, as suggested by subgroup analysis according to different BMI. In the analysis of the different study designs, cholelithiasis was associated with the risk of PCa in case–control studies but not in cohort studies. The possible reasons for these disparities may be the limited number of included studies. In the future, we need better-designed studies to confirm this association.

Cholecystectomy is mainly indicated for individuals with biliary tract infection and pancreatitis, and decreases the incidence of the biliary tract and pancreatic malignancy [26, 27]. However, we did not observe that men who received cholecystectomy therapy were not significantly related to a decreased PCa risk. It was demonstrated that cholecystectomy would impair the enterohepatic circulation of bile acids and might increase oxidative stress and oxidative damage to DNA to stimulate prostate cancer cell growth and prostate carcinogenesis. There were epidemiologic studies that provided evidence about the relationship between cholecystectomy and PCa. Chien-Hua Chen et al. [28] reported data from 72,606 gallstone cases and the investigators assessed that cholecystectomy was linked to increased risk for PCa compared with the non-cholecystectomy group were HR = 1.67(95%CI, 1.45–1.92). Therefore, more studies are worth examining whether other factors affect risk discrepancies.

The underlying mechanism of cholelithiasis might lead to malignancy development still deserves to be explored. The potential mechanisms for the association of cholelithiasis with PCa may be the following. Firstly, prostate cancer may be mediated by the cholesterol metabolism associated with cholelithiasis. Cholesterol was critical for the proliferation of cells, and its synthesis was tightly synchronized to cell cycle progression. Cholesterol-lowering may induce apoptosis in PCa cells progressing through the cell cycle [10, 29–32]. In addition, the progress of PCa depends on the existence of androgen. Cholesterol plays a vital role in androgen synthesis; consequently, it is possible that cholesterol promotes cancer growth [33–36]. Second, microbiota dysbiosis could result in cholelithiasis in the gut and biliary tract [37–39]. Intestinal microbial diversity would influence the number of bacteria causing systemic inflammation and prostate tumorigenesis [40–45]. For example, dysbiosis of the gut microbiome can promote conjugation and recycling of estrogens via secretion of the β-glucuronides enzyme, which results in cell proliferation and tumor development [41, 46]. Finally, the metabolic syndrome also can influence the incidence of PCa and cholelithiasis [8, 14]. Metabolic syndrome is associated with increased cancer mortality and tumor aggressiveness, but the specific mechanism is not well-known. It may affect the level of androgen [47–49].

There are some following strengths: to our knowledge, we firstly comprehensively estimated the association between cholelithiasis and PCa. We rigorously used the GRADE approach to assess the quality of evidence for the main findings. However, there are also several following limitations: firstly, with regard to meta-analysis, we include studies that adjusted or controlled for various risk factors, but some unknown or unmeasured residual confounders cannot be excluded. Secondly, in subgroup analysis, there are only two studies about cholecystectomy. We should be cautious about the results of.cholecystectomy. Last, the association between cholelithiasis and differ-grade PCa may be due to the limited current studies and well-designed studies are required to explore.

## Conclusion

In conclusion, our study supported the associations of cholelithiasis with the increased risk of PCa in European and Asian populations. There was no significant relationship between cholecystectomy therapy and PCa risk. Further cohort studies should be conducted to better identify more mechanisms in the pathogenesis of PCa.

## Data Availability

All data generated or analyzed during this study are included in this manuscript.

## Abbreviations

PRISMA: Preferred reporting items for systematic review and meta-analysis
RR: Relative risk
OR: Odds ratio
HR: Hazard ratio
CI: Confidence interval
CIs: Confidence intervals
NOS: Newcastle–Ottawa scale
PCa: Prostate cancer
BPH: Benign prostatic hyperplasia
